# Graphene Oxide/Chitosan Injectable Composite Hydrogel for Controlled Release of Doxorubicin: An Approach for Enhanced Intratumoral Delivery

**DOI:** 10.3390/nano12234261

**Published:** 2022-11-30

**Authors:** Safaa Eltahir, Reem Al homsi, Jayalakshmi Jagal, Iman Saad Ahmed, Mohamed Haider

**Affiliations:** 1 Department of Pharmaceutics and Pharmaceutical Technology, College of Pharmacy, University of Sharjah, Sharjah 27272, United Arab Emirates; 2 Research Institute of Medical & Health Sciences, University of Sharjah, Sharjah 27272, United Arab Emirates

**Keywords:** graphene oxide, chitosan, composite hydrogel, controlled release, optimization, doxorubicin

## Abstract

Intratumoral (IT) injection of chemotherapeutics into needle-accessible solid tumors can directly localize the anticancer drug in the tumor site, thus increasing its local bioavailability and reducing its undesirable effects compared to systemic administration. In this study, graphene oxide (GO)-based chitosan/β-glycerophosphate (CS/GP) thermosensitive injectable composite hydrogels (CH) were prepared and optimized for the localized controlled delivery of doxorubicin (DOX). A quality-by-design (QbD) approach was used to study the individual and combined effects of several formulation variables to produce optimal DOX-loaded GO/CS/GP CH with predetermined characteristics, including gelation time, injectability, porosity, and swelling capacity. The surface morphology of the optimal formulation (DOX/opt CH), chemical interaction between its ingredients and in vitro release of DOX in comparison to GO-free CS/GP CH were investigated. Cell viability and cellular uptake after treatment with DOX/opt CH were studied on MCF 7, MDB-MB-231 and FaDu cell lines. The statistical analysis of the measured responses revealed significant effects of the concentration of GO, the concentration of CS, and the CS:GP ratio on the physicochemical characteristics of the prepared GO/CS/GP CH. The optimization process showed that DOX-loaded GO/CS/GP CH prepared using 0.1% GO and 1.7% CS at a CS: GO ratio of 3:1 (*v/v*) had the highest desirability value. DOX/opt CH showed a porous microstructure and chemical compatibility between its ingredients. The incorporation of GO resulted in an increase in the ability of the CH matrices to control DOX release in vitro. Finally, cellular characterization showed a time-dependent increase in cytotoxicity and cellular uptake of DOX after treatment with DOX/opt CH. The proposed DOX/opt CH might be considered a promising injectable platform to control the release and increase the local bioavailability of chemotherapeutics in the treatment of solid tumors.

## 1. Introduction

Solid tumors comprise the majority of human cancers and are characterized by a dense mass of malignant cells, poorly structured microvasculature, irregular blood flow, lack of proper lymphatic drainage and high interstitial fluid pressure (IFP) [1,2]. Common treatment strategies include surgery, radiotherapy, chemotherapy and immunotherapy alone or in combination. Tumor resection is considered the most effective approach; however, it is highly invasive and may fail to yield prosperous therapeutic results, particularly in the metastatic stages. Moreover, it is often associated with disfigurement, especially when it involves superficial tumors such as breast cancer and head and neck squamous cell carcinomas (HNSCC) [3,4]. On the other hand, the use of radiotherapy in the treatment of solid tumors is limited by severe toxic side effects that include radiation dermatitis, neurotoxicity, ototoxicity and hematologic toxicity [5]. Parenteral administration of chemotherapeutic agents via intravenous (IV) or intra-arterial routes is limited by their low selectivity, poor biodistribution, fast elimination, and systemic toxicity, especially in high doses [6]. In addition, the administration of anticancer drugs by parenteral routes often fails to deliver a therapeutically effective concentration of chemotherapy that is essential for the eradication of solid tumors. For instance, it has been reported that less than 0.5% of the total dose of paclitaxel is available at the tumor site when administered via intravenous infusion for the treatment of lung cancer [7]. This may be due to the disorganized structure of solid tumors and lack of proper blood perfusion, which hinders the ability of the chemotherapeutic and/or immunotherapeutic agents to effectively reach the core of a large solid tumor. As a result of this repeated exposure to lower-than-lethal doses of chemotherapeutics, malignant cells may develop anticancer drug resistance [8,9,10]. 

Direct intratumoral (IT) injection of chemotherapeutic agents is considered a suitable approach for the treatment of needle-accessible superficial solid tumors such as breast cancer and HNSCC tumors as it increases the local bioavailability of the anticancer agent at the tumor site and reduces its undesirable systemic effects [11]. Furthermore, direct injection of chemotherapeutic agents into solid tumors can allow them to reach poorly perfused tumor regions in adequate and effective concentrations that cannot be achieved through systemic administration due to the independence of cancer vasculature [12]. However, local injection of an anticancer drug solution may result in its diffusion to the surrounding tissue leading to severe damage to healthy cells and an inevitable leakage to the lymphatic and systemic circulation [7]. As such, alternative strategies to sustain the local delivery of chemotherapeutic agents, prolong their retention at tumor sites, reduce their frequency of administration, and minimize their leakage to surrounding tissues are of great importance.

Thermosensitive injectable hydrogel scaffolds that are capable of retaining the drug at the site of injection and controlling its release may be considered an effective approach. At room temperature, these hydrogels exist as free-flowing polymeric solutions that allow for ease of formulation, homogeneous drug distribution and ease of injection. A sol-to-gel transition occurs post-injection and results in the formation of a hydrogel scaffold, which might act as a depot that slowly releases its drug load, thus maintaining constant therapeutic levels in the vicinity of the tumor site for prolonged periods [13,14]. Thermosensitive injectable hydrogel scaffolds composed of natural and synthetic polymers and their combinations have been widely used for localized delivery of chemotherapeutic agents for the treatment of different types of malignant tumors [7,15]. 

Aqueous mixtures of chitosan (CS) and β-Glycerophosphate (GP) have been reported to respond nonlinearly to temperature elevation resulting in the physical crosslinking of the CS polymeric chain and the formation of a hydrogel [16,17]. The sol-to-gel transition temperature depends on the CS:GP ratio, the molecular weight of CS, and the pH and ionic strength of the medium. By changing those factors, the sol-to-gel transition temperature of the CS/GP hydrogel forming solution can be adjusted to occur at body temperature within reasonable time frames. In addition, CS/GP thermosensitive injectable hydrogel scaffolds are biocompatible and biodegradable, thus presenting a practical platform for a variety of biomedical applications [18,19]. Nevertheless, the clinical use of CS/GP thermosensitive hydrogels is still restricted by their batch-to-batch variation and their poor mechanical properties, which limit their ability to retain the formed hydrogel structure in situ and control drug release [20,21]. 

Composite hydrogel (CH) technology has attracted increasing attention as a strategy to improve the mechanical characteristics of hydrogels and, in particular, CS/GP thermosensitive injectable scaffolds [22,23]. The incorporation of metal oxide nanoparticles, carbon nanotubes and nanocellulose fibers was reported to increase the mechanical strength of the CS/GP hydrogel scaffolds and alter their thermal sensitivity, swelling capacity and encapsulation efficiency [15,23,24,25]. Graphene oxide (GO) is a two-dimensional honeycomb nano lattice bearing various oxygen-rich functional groups such as carbonyl, hydroxyl, carboxylic and epoxy groups which explains its remarkable dispersibility in aqueous media. GO nanosheets are also characterized by their enormous surface area, unique optical properties, biocompatibility and chemical stability [26]. When incorporated as a filler in composite hydrogels, GO interacts with protonated polymer chains such as CS and acts as a physical crosslinking agent that alters the physicochemical and mechanical properties of the hydrogel matrix. Several studies reported the use of GO in the preparation of CS-based composite hydrogels (CH), including CS, CS-graft-poly(acrylic acid), poly(N-isopropylacrylamide)/CS copolymer, and CS-based polyacrylamide double network in different drug delivery and tissue engineering applications [21,27,28,29].

In a previous study, we incorporated different concentrations of GO (0.05–0.2%) in CS/GP thermosensitive hydrogels to control the release of bupivacaine. The results showed a considerable improvement in the mechanical properties of the CH and enhancement of the local anesthetic effect of the drug [30]. Other factors, such as the concentration of CS and the CS/GP ratio, were also reported to affect drug loading capacity, drug release, biodegradability and mechanical strength of the hydrogel matrix [25,31,32]. A systematic quality by design (QbD) strategy is required to determine the possible relationship between the different formulation factors and their effect on the crosslinking density and other related properties that affect localized drug delivery from GO-based CS/GP injectable CH. The aim of this work was to prepare, optimize and characterize DOX-loaded GO/CS/GP CH scaffolds as a potential therapeutic approach that might be used to improve the local bioavailability and control drug release in the tumor site following IT injection to accessible solid tumors. A D-optimal response surface design was used to study the effects of the concentration of GO, the concentration of CS and the CS:GP ratio and their interactions as the critical material attributes (CMAs) for the experimental design, on the gelation time, the force required for injection (injectability), degree of porosity, and percentage swelling of the prepared hydrogels. The optimal drug-loaded injectable CH formulation (DOX/opt CH) was further studied for possible chemical interaction between its components, surface morphology and in vitro drug release in comparison to drug-loaded GO-free CS/GP scaffolds. Cell proliferation and cellular uptake of DOX by breast cancer and HNSCC cell lines after treatment with the optimal injectable CH scaffold were also investigated.

## 2. Materials and Methods

### 2.1. Materials

Graphene oxide (GO) was obtained from Graphenea (Donostia, Gipuzkoa, Spain). Doxorubicin hydrochloride salt (DOX, molecular weight: 579.98 Da) was purchased from LC laboratories (Woburn, MA, USA). Chitosan (CS, medium molecular weight 190–310 kDa, deacetylation ≥ 75%), β-glycerophosphate disodium salt hydrate (GP, molecular weight = 216.04 Da), cellulose dialysis membrane (molecular weight cut-off (MWCO) 14 kD), acetic acid, absolute ethanol, Eagle’s minimum essential medium (MEM), Dulbecco’s Modified Eagle medium (DMEM), fetal bovine serum (FBS), penicillin/streptomycin, trypsin/ ethylenediaminetetraacetic acid (EDTA), sodium dodecyl sulfate (SDS) and dimethyl sulfoxide (DMSO) were purchased from Sigma Aldrich (St. Louis, MO, USA). 

### 2.2. Experimental Design

The optimization of drug-loaded GO-based CS/GP composite hydrogels (DOX-loaded GO/CS/GP CH) was carried out using Design-Expert^®^ software (Version 13.0, Stat-Ease Inc., Minneapolis, MN, USA). The design involved investigating the effect of 3 numerical factors (continuous or discrete): the concentration of GO (0–0.1% *w/v*; X_1_), the concentration of CS (1.5–2% *w/v*; X_2_), and the CS:GP ratio (2:1 and 3:1 *v/v*; X_3_), as critical material attributes (CMAs) for the selected critical quality attributes (CQAs). The levels of the independent variables were defined based on their ability to provide maximal design space and allow conceivable processing of the hydrogel formulations (Table 1). Planning the levels of the independent variables and analysis of the output data with the minimal number of experimental runs was carried out using a D-optimal response surface design. The responses measured for the purpose of optimization of DOX-loaded GO/CS/GP CH included gelation time (Y_1_), the force required for injection (Y_2_), degree of porosity (Y_3_), and swelling capacity (Y_4_). The hydrogel formulations were optimized for the measured responses (Y_1_–Y_4_) with the target response set at a gelation time of around 3 min, minimum force injection, the lowest degree of porosity and maximum percentage swelling.

The proposed statistical design generated 19 experimental runs (formulations), including 4 replications (Table 2). All hydrogels were prepared and characterized in random order to increase the predictability of the model and eliminate biased variance. Measurements were carried out in triplicate (n = 3), and the mean ± standard deviation (SD) for each sample was recorded. The responses obtained for the 19 experimental runs were fitted to linear, two-factor interaction (2FI), and quadratic response surface models. Analysis of variance (ANOVA) was used to ensure the significance of the polynomial equations created by the Design Expert^®^ software. The model was statistically validated using p-value, multiple correlation coefficient (R^2^), adjusted multiple correlation coefficient (R^2^-adj), predicted multiple correlation coefficient (R^2^-pred) and adequate precision. Model selection was based on maximized R^2^-adj and R^2^-pred and a signal-to-noise ratio greater than 4 [33,34]. Three-dimensional response surface plots showing the effect of the CMAs on the different responses were created by Design-Expert^®^ software for graphical evaluation of the results and determination of the degree of interactions between the independent variables. Next, the desirability function was applied to anticipate the variables range where the optimized composition would exist based on numerical and graphical analysis. Desirability values closer to zero were considered least desirable, while those closer to 1 were correlated to the desired response.

### 2.3. Preparation of DOX-Loaded GO/CS/GP CH

DOX-loaded GO/CS/GP CH was prepared following the experimental design (Table 2) based on the previously reported method with some modifications [30]. Briefly, GO (0, 5, 10 mg) was added to 6.6 mL or 7.5 mL of 0.1 M acetic acid (S1 and S2, respectively), followed by ultrasonication using a probe ultrasonicator (Q500, Terra Universal, Inc., Fullerton, CA, USA) at 50 MHz for 2 min in an ice bath. Next, 30 mg of DOX was added to each solution under stirring at room temperature until complete dissolution, followed by the addition of 1.5–2 mg of CS. The aqueous mixture was left under stirring overnight at room temperature using a magnetic stirrer. GP aqueous solution (pH = 7) was prepared by dissolving 5 g in 10 mL distilled water followed by slow addition of 3.3 mL and 2.5 mL dropwise to DOX/GO/CS solutions S1 and S2 to yield 2:1 and 3:1 CS/GP (*v/v*) ratios, respectively, under constant stirring on an ice bath. The final concentration of DOX in the pregel solution was 0.3% (*w/v*). In order to avoid premature gelation, the prepared formulations were stored at 4 °C until further testing. DOX-free hydrogel-forming solution was prepared using the same technique to serve as a control.

### 2.4. Physicochemical Characterization of DOX-Loaded GO/CS/GP CH

#### 2.4.1. Gelation Time

The time needed for sol-to-gel transition at 37 °C was determined using the tube inverting method [25]. Briefly, glass vials containing 1 mL of pregel solutions were kept at 37 °C. The vials were inverted every 20 s, and the flow capacity of the samples was assessed. The time at which the tested formulation can no longer flow was recorded for each preparation. All experiments were conducted in triplicates (n = 3), and the data are reported as mean ± SD.

#### 2.4.2. The Force Required for Injection

The force required for injection or injectability of the hydrogel-forming solution was assessed using a TA-XT Plus texture analyzer (Stable Micro Systems, Surrey, UK). The method depends on measuring the force required for mechanical compression of the plunger of a syringe containing the tested material at a set rate [30]. Measurements were carried out at room temperature using 1 mL plastic syringes attached to 21 G needles. Each syringe was cautiously filled with 1 mL of the prepared hydrogel-forming solution to avoid the introduction of air bubbles and then attached to the texture analyzer using the Universal Syringe Rig (A/USR) fixture. The samples were extruded from the syringe at a constant rate of 1 mm/s, and the force required for injection (N) of each hydrogel-forming solution was recorded. All measurements were done in triplicates (n = 3), and the data are reported as mean ± SD.

#### 2.4.3. Degree of Porosity

The degree of porosity of DOX-loaded GO/CS/GP CH was measured using the gas-ethanol replacement method [35]. Briefly, predetermined equal volumes of pregel solutions (n = 3) were incubated at 37 °C for 2 h till complete gelation. The gels were then stored in a −80 °C freezer overnight before being lyophilized using a freeze-dryer (Vir Tis Bench Top Pro, SP Scientific, Warminster, PA, USA) with a condenser temperature of −50 °C and a pressure of 7 × 10^−2^ mbar for 48 h. A known excess volume (10 mL) of absolute ethanol (*E*_1_) was added to the freeze-dried gels. The gels were then stored at room temperature for 10 min for complete wetting, and then the total volume of ethanol and immersed hydrogel was recorded (*E*_2_). Finally, the immersed hydrogel was carefully removed, and the remaining volume of ethanol (*E*_3_) was recorded using a graduated pipette. The degree of porosity of the hydrogels (*p*) was determined using the following equation:

(1) $$p\left(\%\right)=\frac{\left(E_{1}-E_{3}\right)}{\left(E_{2}-E_{3}\right)}\times 100$$

where *E*_1_ − *E*_3_ represents the volume of ethanol entrapped in the pores of the composite hydrogel, while *E*_2_ − *E*_3_ is the volume of the measured composite hydrogel. All measurements were done in triplicates (n = 3), and the results are reported as mean ± SD.

#### 2.4.4. Swelling Capacity

In order to measure the swelling capacity of the composite hydrogels, equal volumes of the pregel solutions were incubated at 37 °C for 2 h for complete gelation, followed by freezing at −80 °C overnight and lyophilization as previously described. The dry weights (H*_d_*) of the freeze-dried CH were recorded, and then the gel matrices were submerged in 10 mL phosphate buffer (pH 7.4) and kept at 37 °C for 24 h. Next, the gels were retrieved and carefully washed with deionized water to discard any adsorbed ions, then carefully blotted dry using filter paper. The wet weight (H*_w_*) of each sample was accurately measured, and the swelling ratio (*q*), defined as the fractional increase in the weight of the hydrogel as a result of water absorption, was calculated based on the following equation [36,37]:

(2) $$q=\frac{H_{w}-H_{d}}{H_{d}}\times 100$$

All measurements were performed in triplicates, and the results are reported as mean *q* ± SD. 

### 2.5. Fourier Transform Infrared (FT-IR) Spectroscopy

FT-IR analyses were conducted to identify any significant chemical interactions between the various components of the hydrogel. Powders of DOX, GO, CS, and GP and the lyophilized optimal formulation of DOX-loaded GO/CS/GP CH (DOX/opt CH) were mixed individually with 100 mg of potassium bromide at a ratio of 1:100 and then pressed into a round thin transparent disc using a hydrostatic pressure of 10,000–15,000 pounds per square inch. The IR spectrum of each sample was determined over a wavelength range from 4000 to 400 cm^−1^ at a resolution of 4 cm^−1^ using an FT-IR spectrophotometer (JASCO FTIR 6300, Jasco, Easton, MD, USA). 

### 2.6. Scanning Electron Microscopy 

The shape, surface structural features and cross-sectional view of the freeze-dried optimal formulation DOX/opt CH were investigated using scanning electron microscopy (SEM). The samples were lyophilized as previously described, then treated with liquid nitrogen and fractured to prepare the cross-sections. Each specimen was then fixed on a metal stub using a conductive tape and sputter-coated with a Gold–Palladium (80–20%) in a high vacuum using a Mini Sputter Coater (Q150TS Quorum Technologies, East Sussex, UK). The gold-coated samples were scanned using a Thermo Scientific Apreo SEM (FEI Company, Hillsboro, OR, USA), and photomicrographs were captured at 15 kV accelerating voltage and a working distance of 17 mm. SEM images of the samples were compared to GO-free hydrogels prepared using the same CS concentration, drug content and CS/GP ratio to study the effect of adding GO on the optimized hydrogel formulation. 

### 2.7. In Vitro Release Studies

The in vitro release studies were performed to compare the release of DOX from the optimal formulation (DOX/opt CH) relative to GO-free DOX-loaded hydrogel (DOX/CS/GP) and free DOX solution. The in vitro release experiments were carried out using a modified diffusion method [32,38]. Briefly, 3 mL of the hydrogels or drug solution were loaded in cellulose dialysis membrane (MWCO 14 kDa). The membranes were then sealed and inserted in glass vials containing 10 mL of phosphate buffer (pH 7.4). Next, the vials were tightly closed and kept in a shaking water bath (OLS Aqua Pro, Grant Instruments, Cambridgeshire, UK) at 37 ± 0.5 °C and 100 rpm. At specific time intervals (0.25, 0.5, 1, 2, 4, 6, 8 and 12 h; 1, 2, 3, 4, 5, 6, 7, 14, 21, and 28 days), 1 mL of the dialysis medium was withdrawn and immediately replaced with an equal volume of fresh buffer. The percentage cumulative release of DOX from the hydrogels or drug solution was determined via UV spectroscopy at 480 nm using a Synergy™ HTX microplate reader (BioTek, Winooski, VT, USA). A standard curve of DOX in phosphate buffer (pH = 7.4) was generated over the range of (0.001–1 mg/mL) and was used to convert absorbance to concentration (Supplementary Figure S1). The in vitro release tests were performed in triplicates (n = 3), and the results are reported as mean ± SD.

The kinetics of drug release from the tested hydrogels were determined by fitting the obtained data to zero-, first-, and second-order kinetics equations as well as the Higuchi diffusion equation [39,40]. Zero-order kinetics were determined using the equation:

*C_t_* = *C_o_* − *kt* (3)

where *C_t_* is the concentration of the drug at time *t*, *C_o_* is the initial drug concentration, and *k* is the apparent release rate constant. The following linear regression equation was used for computing first-order kinetics:

*lnC_t_* = *lnC_o_* − *kt* (4)

while second-order kinetics were calculated using the following equation:

(5) $$\frac{1}{C_{t}}=\frac{1}{C_{o}}+kt$$

Finally, drug release following the Higuchi model was examined using the following equation:

*Q* = *kt*^0.5^ (6)

where *Q* represents the fraction of drug released in time *t* while *k* is the Higuchi release rate constant.

### 2.8. Cell Culture

Human breast adenocarcinoma cell lines (MCF 7 and MDB-MB-231) and human pharyngeal squamous carcinoma cell lines (FaDu) were acquired from American Type Culture Collection (ATCC; Manassas, VA, USA). MCF 7 and MDB-MB-231 cells were cultured in DMEM, while FaDu cells were cultured in MEM. All media were supplemented with 10% heat-inactivated FBS, 100 U/mL penicillin and 100 μg/mL streptomycin. The cells were incubated under humidified air and 5% carbon dioxide at 37 °C. 

### 2.9. Cytotoxicity and IC_50_ Studies

The effect of DOX/opt CH on cell viability was investigated using MCF 7, MDA-MB-231 and FaDu cell lines. The cells were seeded in 96-well plates (Corning, Sigma-Aldrich Co., St. Louis, MO, USA) at a seeding density of 5 × 10^3^ cells/well and cultured overnight for initial attachment. On the first day of the study, the cells were incubated with different volumes of the optimal DOX-loaded hydrogel-forming solution containing 3.125–100 μM of the drug, 0.625–10 μM of free drug solution or 100 μL of DOX-free optimal formulation (Blank Opt CH) placed in transwells above the cultured cells. In addition, cells were treated with fresh culture media, DMSO (0.4% *v/v*) and SDS (1% *w/v*) as controls. After 24 h, 48 h and 72 h, the antiproliferative activity of each treatment was assessed using MTT (3-(4,5-dimethylthiazol-2-yl)-2,5-diphenyltetrazolium bromide) assay kit. At each time point, the cells were washed with PBS (pH 7.4), followed by the addition of 10 µL of 5 mg/mL MTT dye solution. After 4 h of incubation at 37 °C, the culture media containing MTT were removed, 100 μL DMSO was added to each well, and the plates were shaken for 20 min. The optical intensity was measured at 570 nm using a Synergy™ HTX microplate reader (BioTek, Winooski, VT, USA), and the cell viability was determined using the following equation [41]:

(7) $$\mathrm{Cell}\;\mathrm{viability}\;\%=\frac{\mathrm{Sample}\;\mathrm{mean}\;\mathrm{optical}\;\mathrm{density}}{\mathrm{Negative}\;\mathrm{control}\;\mathrm{mean}\;\mathrm{optical}\;\mathrm{density}}\times 100$$

All experiments were carried out in triplicates, and the results are reported as mean measurement ± SD. For the determination of the half-maximal inhibitory concentration (IC_50_), the cells were treated with 3.125–100 μM DOX solution or DOX/opt CH for 24 h, 48 h and 72 h following the same procedure and the obtained results were statistically analyzed using Prism version 9.2.0 (GraphPad Software, San Diego, CA, USA).

### 2.10. Cellular Uptake

In vitro cellular uptake of DOX after treatment with DOX/opt CH was assessed using confocal laser scanning microscopy and quantified using flow cytometry. For fluorescent microscopy, MCF 7, MDA-MB-231 and FaDu cell lines were seeded into 24-well culture plates and grown until reaching 70% cellular confluency. The cells were then treated with free DOX solution (10 μM) or DOX/opt CH (10 μM) placed in transwells above the cultured cells as described earlier and incubated at 37 °C for 24 h followed by washing three times with PBS (pH 7.4). Next, the cells were fixed with 5% paraformaldehyde and incubated at room temperature for 30 min. Finally, the cells were stained with DAPI-containing mounting medium and examined using a confocal microscope (Nikon eclipse Ti Melville, New York, NY, USA). 

For the determination of fluorescence intensity using flow cytometry, MCF 7, MDA-MB-231 and FaDu cells were treated with free DOX solution (10 μM), DOX/opt CH (10 μM), or growth media as a control for 24 h. At 70% confluency, the cells were treated with trypsin/EDTA and diluted with 500 µL PBS (pH 7.4). The samples were acquired by BD FACSAria III flow cytometer (BD Biosciences, San Jose, CA, USA). The fluorescent intensity of each sample was assessed via BD FACSDiva software (BD Biosciences, San Jose, CA, USA) using standard fluidics, optical and electronic configuration. 

### 2.11. Statistical Analysis

All experiments and measurements were performed in triplicates, and the reported values are presented as mean ± SD. One-tailed unpaired student’s *t*-test was used for comparisons between 2 groups, while a 1-way analysis of variance (ANOVA) was utilized for testing the equality of several means. Statistical analysis was carried out using software Prism version 9.2.0 (GraphPad Software, San Diego, CA, USA) and Design-Expert^®^ software (Version 13.0, Stat-Ease Inc., Minneapolis, MN, USA). A *p*-value of ≤0.05 was considered statistically significant.

## 3. Results and Discussion 

### 3.1. Preparation of DOX-Loaded GO/CS/GP CH

The use of the ionic interaction technique for crosslinking in-situ gelling hydrogels has drawn special attention because it is simple and does not involve the addition of chemical crosslinkers. Therefore, physical crosslinking has been widely used to prepare biodegradable hydrogel scaffolds with low toxicity for controlled drug release and tissue engineering applications [25,30,42,43,44]. 

Physical crosslinking of cationic CS chains through ionic interactions involves the use of multivalent counter-ions. In this study, DOX-loaded injectable CH was successfully prepared using the ionic interaction technique. The addition of GO- and GP-bearing anionic carboxylic and phosphate groups, respectively, to CS below its critical solution temperature (CST), results in the neutralization of the positively charged amino groups present on its polymeric chains. Although these ionic interactions result in the reduction of inter-chain repulsion, the hydroxyl groups on GO and GP keep the polymer chains hydrated and maintain their solubility in water. However, an increase in the surrounding temperature might lead to the loss of water of hydration, increase hydrogen bonding and hydrophobic interactions between the polymer chains and result in hydrogel formation [16,17].

### 3.2. Optimization of DOX-Loaded GO/CS/GP CH 

The current approach for pharmaceutical product design involves establishing an initial list of quality requirements known as a quality target product profile (QTPP). This list represents the prospective summary of the properties required to ensure the desired quality, taking into consideration the safety and efficacy of the product [45]. Moreover, this list forms the basis for the selection of critical quality attributes (CQAs) for the product. 

QTPP was established considering the quality characteristics of a DOX-loaded injectable composite hydrogel formulation intended for IT injection and capable of controlling the release of the incorporated active, thus enhancing its cellular uptake in cancer cells and increasing its local bioavailability in order to achieve the optimal efficacy (Table 3). The optimization of the CMAs and critical process parameters (CPPs) that affect the CQAs of the hydrogel formulation to permit easy handling and injection of the hydrogel into the target site and control the release of the drug represents a great challenge in the design of CH for IT injection [46].

In this study, 19 experimental runs were performed for the optimization of DOX-loaded GO/CS/GP CH as determined by the D-optimal response surface design, including four replications, each prepared under the same conditions to ensure the precision of the statistical model. Moreover, the selected formulation variables and measured responses were set up based on past formulation development reported in earlier studies [25,30,31,32]. Table 2 summarizes the composition and characteristics of the 19 hydrogel formulations prepared using this design.

The obtained responses were fitted to linear, 2FI, quadratic and cubic models using Design Expert^®^ software. Statistical analysis of the experimental design showed that the force required for injection was best fitted to the two FI models, while gelation time, degree of porosity, and swelling capacity were best fitted to the quadratic model (Table 4). The calculated differences between the Pred-R^2^ and Adj-R^2^ values for all responses were less than 0.2, indicating consistency between the predicted and the experimental data and revealing that the selected models effectively predict all response values. Moreover, the adequate precision values measuring the signal-to-noise ratio in the design were higher than four for all responses, which confirms that the selected models are suitable for navigating the design space.

The analysis of the data yielded the following second-order polynomial equation:

Y = βo + β_1_X_1_ + β_2_X_2_ + β_3_X_3_ + β_12_X_1_X_2_ + β_13_X_1_X_3_ + β_23_X_2_X_3_ + β_11_X^2^_1_ + β_22_X^2^_2_ + β_33_X^2^_3_ (8)

where Y is the measured response; β_o_ is the intercept coefficient; β_1_, β_2_, β_3_, …, β_33_ are the regression coefficients; X_1_, X_2_, X_3_, are the studied formulation factors at the specified levels; X_1_X_2_, X_1_X_3_, X_2_X_3_ are the interaction terms while X_1_^2^, X_2_^2^, X_3_^2^ represent the quadratic terms. The equation can be used to predict the response values and to emphasize the relative effect of the formulation factors by analyzing and comparing their regression coefficients. The significance of the terms of the regression model (coefficients) was assessed based on the probability value (*p*-value), where a *p*-value less than 0.05 means that the equation of the selected model can be considered statistically significant [47]. 

### 3.3. Effect on the Gelation Time

The time needed for sol-to-gel transition at body temperature is a fundamental CQA for hydrogels intended for IT injection of chemotherapeutic agents. It is considered an essential feature as it affects the proper handling, ease of injection, and formation of a scaffold that is capable of controlling the release of the anticancer drug at the tumor site [18]. Gelation time also affects the diffusion of the drug to the surrounding tissues leading to severe damage to healthy cells and an inevitable leakage to the lymphatic and systemic circulation. Initially, the prepared hydrogels should be in their liquid state at room temperature and then transformed to gel shortly after injection at body temperature. Faster or slower gelation kinetics may have an effect on the syringeability of the hydrogel-forming solution as well as the characteristics of the matrix formed in situ, which could significantly affect the drug release and permeation into the surrounding tissues with possible leakage to the systemic circulation [48,49]. 

**Figure 1:**
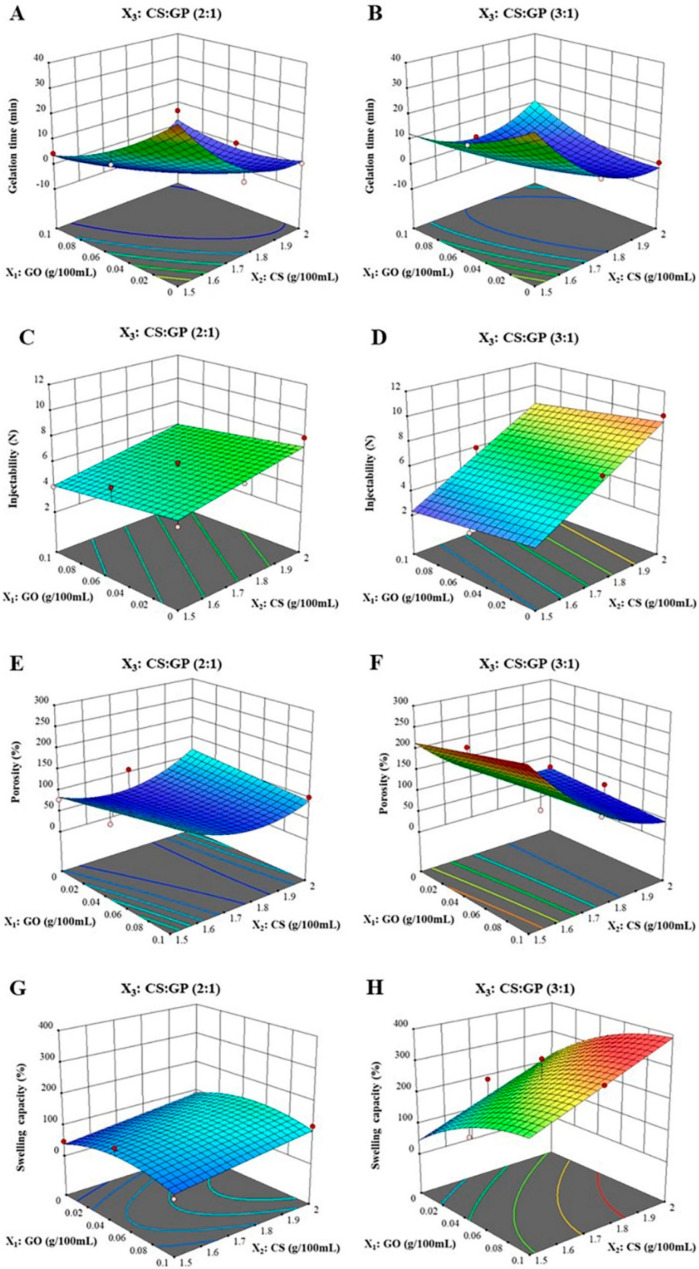
Response surface plots for the effects of GO concentration (X_1_), CS concentration (X_2_) and CS:GP ratio (X_3_) on the gelation time (**A**,**B**); injectability (**C**,**D**); degree of porosity (**E**,**F**) and swelling capacity (**G**,**H**).

The average gelation time for the prepared composite hydrogels at 37 °C ranged from 0.17 to 36.67 min (Table 2). The observed broad gelation time span indicated that the studied CMAs are critical and have a considerable effect on the analyzed response variables. The statistical analysis of the measured gelation time for the 19 experimental runs showed that the concentration of CS (X_2_) had the most significant main effect on the time required for sol-to-gel transition (*p* < 0.0001; Figure 1, Table 4). The concentration of GO (X_1_) also significantly affected the gelation time (*p* = 0.002), while the CS:GP ratio (X_3_) had no effect. Several 2-FI effects between the studied factors at their respective levels on gelation time were observed, indicating the complexity of the statistical model for gelation time. The most significant 2-FI effect was between the GO and CS concentrations (X_1_X_2_, *p* < 0.0001), while a less significant 2-FI was observed between the GO concentration and CS:GP ratio (X_1_X_2_, *p* = 0.0236). The gelation time equation obtained from the analysis was:

Y_1_ = −1.69 − 3.99X_1_ − 8.01X_2_ + 1.49X_3_ + 7.34X_1_X_2_ + 2.84X_1_X_3_ + 3.85X_1_^2^ + 9.28X_2_^2^ (9)

The response surface plots and the quadratic equation showed an indirect relationship between X_1_ and X_2_ and the average gelation time. Faster sol-to-gel transition or shorter gelation times were obtained upon increasing the concentration of CS. This could be due to an increase in polymer-polymer hydrophobic interactions and chain entanglements [16,25,50]. The observed shorter gelation time associated with an increase in the concentration of GO is in correlation with previous studies showing a similar pattern upon the incorporation of small amounts of GO [21,30,51]. This might be due to the combined effects of electrostatic interactions between the carboxylic groups of GO and the amino groups of CS chains and hydrogen bond formation between the oxygen-containing functional groups of GO and the surrounding water molecules. Together, both factors can lead to faster neutralization of the polymer chains and accelerate their dehydration and precipitation into a hydrogel matrix. Interestingly, both X_1_ and X_2_ showed a direct quadratic effect on gelation time. This means that at higher concentrations of CS and GO, longer gelation times or slower sol-to-gel transitions were obtained. At higher concentrations, GO nanosheets may start to form their own network, which acts as a physical barrier for CS chain interaction and prevents matrix formation [52]. This indicates that GO concentration is a critical factor in determining adequate sol-to-gel transition rate for the studied CH formulations.

### 3.4. Effect on the Force Required for Injection 

The administration of injectable hydrogels involves the transfer of the hydrogel-forming solution to the injected target site for subsequent gel formation through an injection device. Therefore, the force required to discharge the injected system through the syringe needle, or injectability, is an essential CQA that ensures the uniformity of flow and ease of injection. It may serve as a guide for the optimization of injectable hydrogel formulations to ensure patient comfort and guarantee safety [53,54]. Factors affecting the injectability of physically crosslinked hydrogels include changes in temperature, pH, ionic strength, molecular weight and concentration of the polymer, crosslinking density and concentration of the counter ions [55,56]. 

Generally, an injectability value of less than 30 N is considered reasonable for ease of injection using 21 G needles [57]. The mean force required for injection was in the range of 2.5 N–10.1 N for the 19 experimental formulations indicating the significant impact of the studied factors on the measured response and the suitable injectability of all prepared pregel solutions. The statistical analysis showed that the concentration of CS (X_2_) had the most significant effect on the force required for injection (*p* < 0.0001), while GO concentration played a less significant role (*p* = 0.0078; Figure 1, Table 4). The analysis also showed a significant 2-FI effect between the CS concentration and the CS:GP ratio (X_2_X_3_, *p* < 0.0001). The polynomial equation describing the force required for the injection of the prepared pregel solutions was:

Y_2_ = 5.84 − 0.5776X_1_ + 2.01X_2_ + 0.142X_3_ + 1.05X_2_X_3_ (10)

The response surface plots and the linear equation revealed a direct relationship between the concentration of CS and/or the CS:GP ratio and their interaction on the force required to inject the pregel solutions. The statistical analysis demonstrated that a higher force is required for injection as the concentration of CS and/or the CS:GP ratio is increased. Previous studies reported similar results upon examining the effect of the concentrations of the polymer and counter ions on the crosslinking density of physically crosslinked polymers and their injectability [48,55]. On the other, X_1_ showed an opposite effect on the injectability of the pregel solution in which a decrease in the force required for injection was associated with the increase in the concentration of GO and hence facilitating the administration process. The incorporation of GO sheets alters the network structure of the CS/GP hydrogels, weakens the interaction between the polymeric chains and reduces the resistance of the matrix to shear forces. Additionally, increasing the GO concentration might result in more surface interactions between its 2D sheets and promotes their lubricating and gliding features, which leads to a further reduction in the frictional forces between the polymeric chains, thus enhancing hydrogel injectability [30,58]. This further confirms the significant role of GO and the importance of optimizing its concentration to enhance the mechanical performance of the CH scaffolds.

### 3.5. Effect on the Degree of Porosity 

The porosity of the hydrogel is a CQA that plays a significant role in the permeation of water in and out of the matrix and in controlling the rate of drug efflux. In addition, the degree of porosity is mainly affected by the crosslinking density between the polymeric chains forming their matrices which in turn affects the mechanical properties of the hydrogel scaffold and its rate of biodegradation or matrix erosion [30,59]. Loading drug molecules onto hydrogel scaffolds with high crosslinking density and low degree of porosity results in reduced water uptake and slower drug release [60,61]. 

Table 2 shows a wide range in the measured porosity among the 19 experimental runs, which reflects the complexity of the statistical model for the degree of porosity. The statistical analysis showed that the concentration of CS (X_2_) had the most significant effect (*p* < 0.0001), followed by the CS:GP ratio (X_3_, *p* = 0.0064; Figure 1, Table 4). The analysis also showed a significant 2-FI effect between CS concentration and CS:GP ratio (X_2_X_3_, *p* < 0.0001). The equation describing the effect of the tested CMAs on the degree of porosity of the hydrogels was:

Y_3_ = 85.58 + 6.11X_1_ − 46.36X_2_ + 20.36X_3_ − 19.03X_1_X_2_ − 44.52X_2_X_3_ + 42.87X_2_^2^ (11)

It could be concluded from the obtained response surface plots and linear equation that an increase in the concentration of CS was associated with a decrease in the degree of porosity of the hydrogels. This might be due to an increase in the crosslinking density associated with the occurrence of more hydrophobic interactions between the polymer chains at higher CS concentrations, thus generating a more compact CH structure with a lower water affinity (concentration-induced gelation). On the other hand, an increase in the ratio of CS:GP (X_3_) was associated with an increase in the porosity of the CH formulations. At high CS:GP ratios, there is a decrease in the available phosphate groups of GP that interact with the more abundant amine groups of CS. Therefore, the ionic crosslinking between CS and GP decreases while the electrostatic repulsion between chitosan molecules increases compared to a lower CS:GP ratio with the subsequent formation of a less coherent gel with increased porosity [62]. 

### 3.6. Effect on the Swelling Capacity

The extent of water uptake is an essential quality attribute for the formulation of hydrogels for drug delivery applications as it controls the swelling behavior of the scaffolds, which in turn affects the rate of drug dissolution, drug diffusion and drug release from the gel matrix. The swelling capacity of the hydrogel scaffold is a balance between the forces which constrain the distortion of the structure of its polymeric network and the osmotic pressure that results in water uptake [63]. A low swelling capacity is usually associated with a stiffer hydrogel matrix and a more rigid network [32]. Several factors, including crosslinking density, nature and concentration of the polymer and counter ions, pH, temperature and ionic strength of the surrounding medium, might alter the swelling behavior of hydrogel [62,64,65]. 

The average swelling capacity of the prepared DOX-loaded GO/CS/GP CH ranged from 41.27% to 335.66%, as demonstrated in Table 2. The statistical analysis showed that the CS:GP ratio (X_3_) had the most significant effect (*p* < 0.0001) on the swelling capacity of the prepared CH scaffolds. The concentration of GO (X_1_) and CS (X_2_) also significantly affected the swelling capacity (*p* = 0.001, *p* = 0.0001, respectively). In addition, the analysis showed 2-FI effects between the studied factors at their respective levels on the swelling capacity of the prepared formulations. The most significant 2-FI effect was between the GO and CS concentrations (X_1_X_3_, *p* = 0.0043), while less convincing evidence of a 2-FI was observed between the CS concentration and CS:GP ratio (X_2_X_3_, *p* = 0.0124), indicating the complexity of the statistical model for swelling capacity measurements. The equation describing the swelling capacity of the composite hydrogels was:

Y_4_ = 181.25 + 45.80X_1_ + 57.90X_2_ + 72.14X_3_ + 37.29X_1_X_3_ + 31.53X_2_X_3_ − 35.90X_1_^2^ (12)

The quadratic equation and response surface plots showed a direct relationship between the concentration of GO and CS, the CS:GP ratio, and the swelling capacity of the composite hydrogels. (Figure 1 and Table 4). The noticed increase in swelling capacity can be justified by the hydrophilic nature of both GO and CS. Oxygen-containing functional groups on GO 2D nanosheets and CS polymeric chains enhance water uptake through the formation of hydrogen bonds. In addition, electrostatic interaction between imine/amino groups of CS and water molecules might occur upon mechanical relaxation of the polymer chains due to the hydration of the dry CH scaffolds [66]. The results and statistical analysis further confirm the significant role of the selected CMAs and the importance of optimization of formulation parameters to enhance the mechanical characteristics of the CH scaffolds.

### 3.7. Selection of the Optimal DOX-Loaded GO/CS/GP CH Formulation 

**Figure 2:**
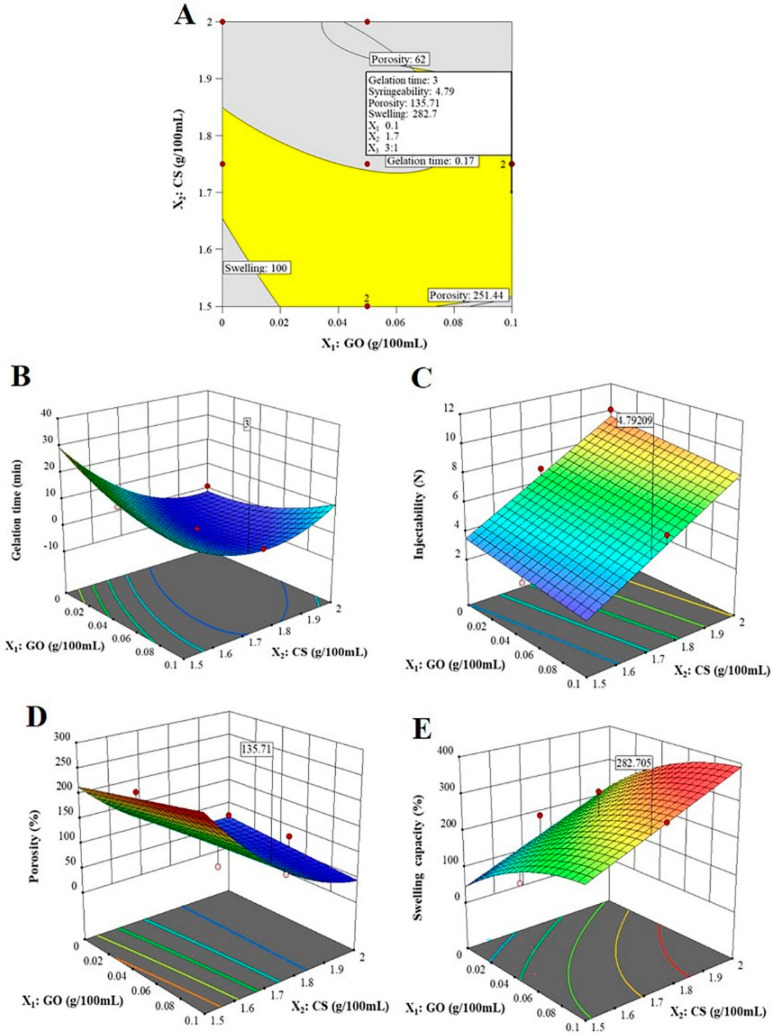
Optimization of DOX-loaded GO/CS/GP CH showing overlay plot for the design space (**A**) and response surface plots for the predicted gelation time (**B**), injectability (**C**), degree of porosity (**D**) and swelling capacity (**E**).

The response surface analysis and the design space in the overlay plot of the D-optimal surface design were used for the prediction of the optimum levels of the independent variables for the optimal formulation of DOX-loaded GO/CS/GP CH (Figure 2). The optimal formulation was coded as DOX/opt CH. The preparation of any formulation within the acceptable design space depicted by the yellow region will result in a CH formulation possessing the target criteria set for the optimization. This included sol-to-gel transition in 3 min at 37 °C to allow proper handling and fast post-injection gel deposition in situ, the minimal force required for injection for ease of injection and patient’s comfort, the minimal degree of porosity, and the maximum swelling capacity to control drug release and increase its retention and residence time at the site of injection. The optimization process involved all responses simultaneously, with the highest priority given to gelation time and ease of injection, followed by the degree of porosity and swelling capacity. The formulation with the highest desirability value (0.777) was obtained using 0.1% GO, 1.7% CS and a CS:GP ratio of 3:1 (*v/v*; Table 5). The observed values for the optimal formulation (DOX/opt CH) were similar to those predicted using 2FI and quadratic equations for all measured responses. Statistical analysis of the results confirmed the validity of the model and concluded that the optimization DOX-loaded GO/CS/GP CH, guided by QbD, provided a formulation with suitable gelation time, injectability, degree of porosity and swelling capacity for possible delivery of DOX by IT injection. 

### 3.8. Physico-Chemical Characterization of DOX/opt GH

#### 3.8.1. FT-IR Spectroscopy

**Figure 3:**
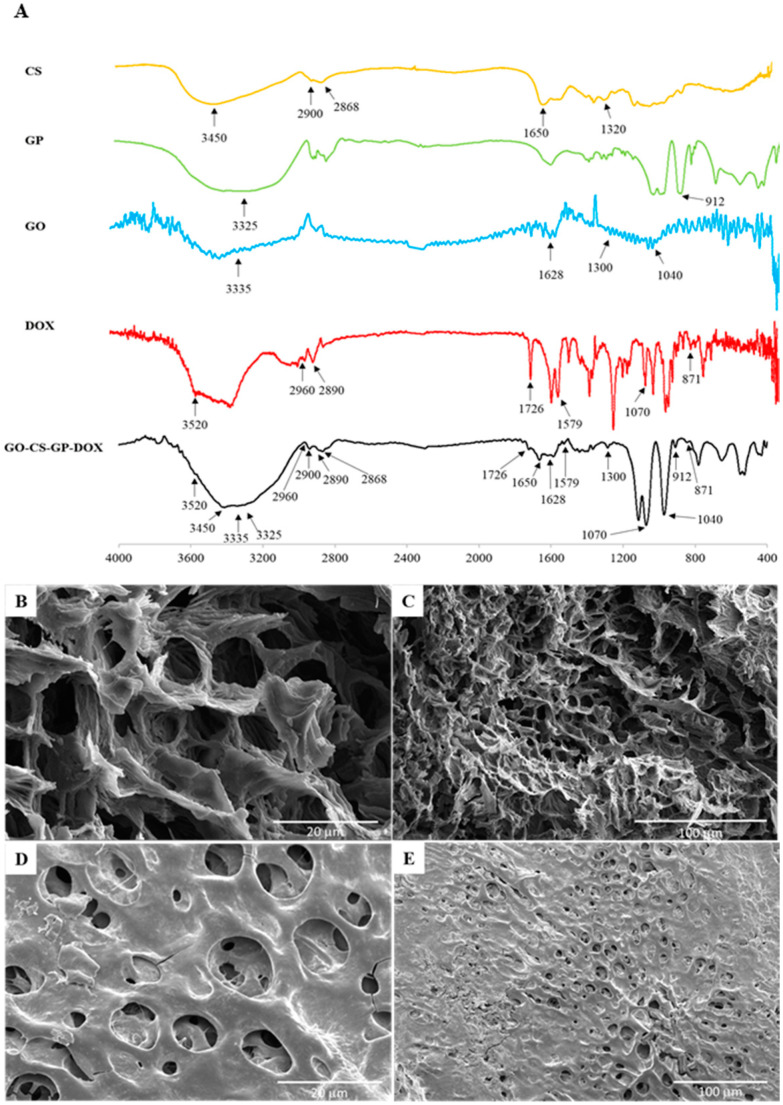
Structural analysis of DOX/opt CH showing FT-IR spectra of the components of the hydrogel and the lyophilized optimal formulation (**A**) and SEM micrographs in the absence of GO (**B**,**C**) and in the presence of GO (**D**,**E**). Scale bars represent 20 μm (**B**,**D**) and 100 μm (**C**,**E**).

The FT-IR spectra of DOX, GO, CS, GP and the lyophilized DOX/opt CH are shown in Figure 3A. CS spectrum showed characteristic stretching vibration of amide C=O at 1650 cm^−1^ and alcohol C-OH at 3200–3600 cm^−1^. GP showed vibration bands at 912 cm^−1^ and 1030 cm^−1^ representing aliphatic phosphate group stretching and at 3200–3600 cm^−1^ representing alcohol C-OH group stretching. The spectrum of GO showed several characteristic stretching vibrations at 1050–1250 cm^−1^ for C-O-C, at 1628 cm^−1^ for carboxylic C=O and at 3200–3600 cm^−1^, typical for C-OH. Strong characteristic IR vibration bands associated with DOX structure were observed, including C-O-C at 1070 cm^−1^, aromatic C=C at 1579 cm^−1^, carbonyl C=O at 1726 cm^–1^, alcohol C-OH at 3200–3600 cm^−1^ and N–H stretch at 3520 cm^−1^. The individual IR spectra for all ingredients used in the fabrication of DOX/opt CH were in correlation with previously reported values [30,67]. The IR spectrum of the lyophilized DOX/opt CH depicted all the characteristic vibration bands of the anticancer agent as well as the other ingredients used in its formulation. The decrease in the intensity and the small shift in the positions of some peaks in the IR spectrum of the optimal formulation could be attributed to the electrostatic interaction and hydrogen bonding formed between the different components of the CH scaffold [30,68].

#### 3.8.2. Microstructure Analysis

The microstructure of a hydrogel is considered an important feature due to its direct effect on the shape, pore size, and free volume of the scaffold [20,69]. The shape and surface morphology of DOX/opt CH with and without GO were investigated using SEM imaging to study the effect of the addition of GO on the organization of the hydrogel matrix. In the absence of GO, the scaffold exhibited a highly porous interconnected microstructure characterized by its rough surface (Figure 3B,C). On the other hand, DOX/opt CH containing GO was characterized by its smooth surface and spherical, small and more uniform pores (Figure 3D,E). The change in the hydrogel topography indicates that the addition of GO can affect the crosslinking of CS chains in the prepared scaffolds and modify their water uptake capacity and ability to entrap the drug and control its release in situ after injection. 

#### 3.8.3. In-Vitro Release Studies

**Figure 4:**
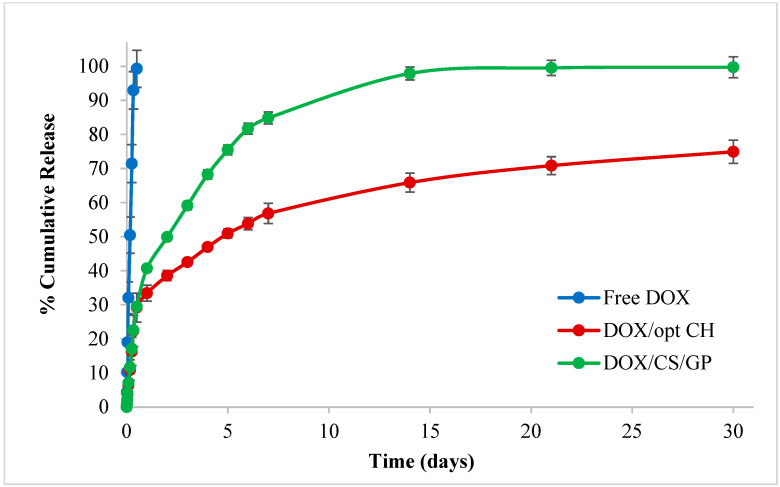
In vitro release studies showing the release of DOX from the optimal formulation (DOX/opt CH) and GO-free DOX-loaded hydrogel (DOX/CS/GP) over 28 days at 37 °C in comparison to free DOX solution. Data points are expressed as mean ± SD (n = 3).

The release of DOX from the DOX/opt CH was compared to its release from an equivalent GO-free hydrogel (DOX/CS/GP) containing a similar CS concentration and CS:GP ratio to investigate the effect of GO on the release of the incorporated drug (Figure 4). Both hydrogels showed a characteristic biphasic sustained release pattern for the drug over 28 days compared to the free DOX solution that was released entirely within 12 h. The first phase was characterized by a faster rate of drug release, where up to 33% and 40% of DOX were released during the first day from DOX/opt CH and DOX/CS/GP, respectively. During the second phase, a slower rate of drug release was observed from DOX/opt CH compared to DOX/CS/GP, where GO-free hydrogels showed complete release of DOX after 28 days, while the DOX/opt CH released only 74% of the drug during the same period. 

The release kinetics best fitted the Higuchi model equation for both DOX/opt CH and DOX/CS/GP with R^2^ = 0.88 and 0.87, respectively, suggesting that the drug release followed a diffusion-controlled pattern in correlation with previous studies using GO-based CH and CS/GP hydrogel scaffolds showing a similar mechanism of drug release [30,32,70,71]. The time required for the drug molecules to diffuse through the monolithic matrix is proportional to the square of the distance traveled by those molecules to the surface and is inversely proportional to the diffusion coefficient of the drug. This can possibly explain the observed biphasic release pattern of DOX as drug molecules located near the surface of the hydrogel matrix are released faster and results in an initial burst release, while those located deeper within the hydrogel matrix take a longer time to come to the surface of the hydrogel before being released.

The release of actives from thermosensitive injectable hydrogel matrices is affected by many factors, including the time required for sol-to-gel transition, porosity, and swelling capacity of the scaffolds. For example, a faster rate of gel formation might result in a better entrapment of the drug in situ following IT injection and a decrease in its initial burst release. Hydrogel scaffolds with higher degrees of crosslinking and lower degrees of porosity are characterized by limited water uptake, drug diffusion and slower drug release. An increase in the swelling capacity of the hydrogel means a more hydrophilic microenvironment with improved water uptake capacity resulting in faster drug dissolution, diffusion and subsequent release [30,32,36,59]. The incorporation of GO resulted in a faster gelation time and better entrapment of the drug, which reduced the initial burst release. In addition, physical interactions between GO nanosheets and CS polymeric chains increased the crosslinking density of the matrix through the formation of three different networks GO-GO, CS-CS and GO-CS. These hydrophilic networks allowed water uptake but acted as physical barriers that further hindered the drug diffusion and subsequent release, therefore, establishing better control over the release of DOX from the optimized CH scaffolds [21,30]. 

### 3.9. Cytotoxicity and IC50 Studies

**Figure 5:**
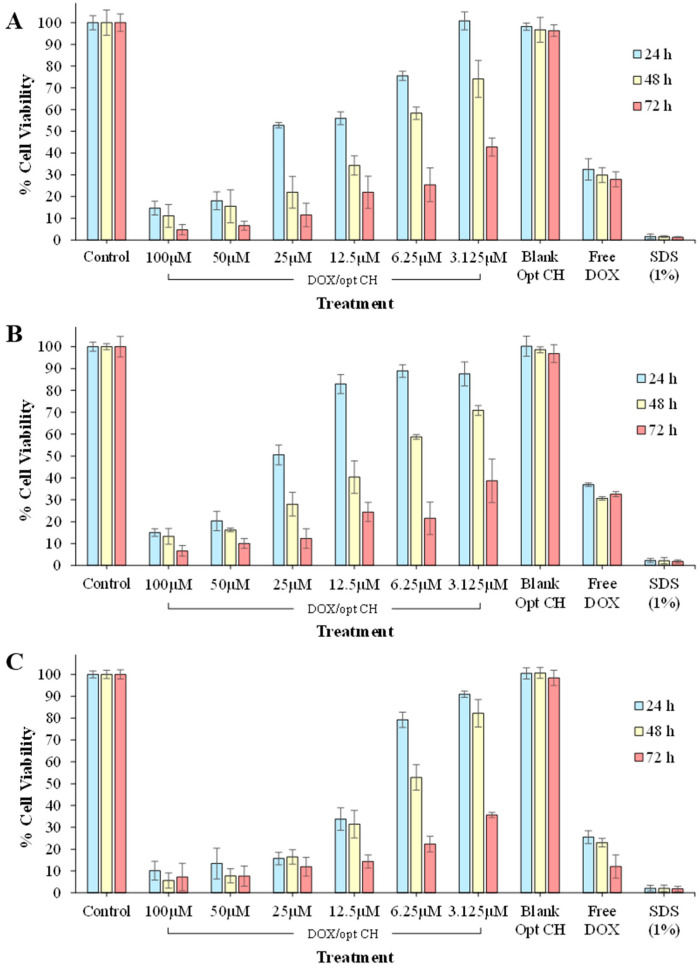
Determination of % cell viability using (**A**) MCF 7, (**B**) MDA-MB-231 and (**C**) FaDu cell lines after treatment with different amounts of drug-loaded optimal formulation (DOX/opt CH), free drug solution (free DOX) and drug-free optimal formulation (Blank Opt CH) for 24 h, 48 h, and 72 h. Data points are expressed as mean ± SD (n = 3).

The cytotoxicity of the DOX/opt CH relative to free DOX solution and DOX-free optimal formulation (Blank Opt CH) was determined at different concentrations of DOX against MCF 7, MDA-MB-231 and FaDu cells using in vitro MTT assay (Figure 5). The biocompatibility and toxicity of GO remain controversial as some studies showed that GO is cyto-friendly while others reported negative biological responses and toxicity [72,73]. Cells treated with Blank Opt CH showed no significant decrease in percentage cell viability compared to the control group indicating the biocompatibility of the optimal CH formulation as the ingredients used in its fabrication did not elicit a cytotoxic effect against the tested cells in vitro. The results are in good correlation with previous studies that showed the biocompatibility of CS/GP CH scaffolds containing 0.5% *w/v* GO with mouse pre-osteoblast cell line and scaffolds prepared using CS, gelatin and GO (0.25% *w/v*) with rat osteoprogenitor cells [21,66].

The percentage of cell viability after treatment with DOX/opt CH was both dose- and time-dependent. Treatment of MCF 7 cells with DOX/opt CH at a low DOX concentration (3.125 µM) resulted in 87% cell viability after 24 h. These percentages further decreased to 71% and 38% when MCF 7 cells were treated for 48 h and 72 h, respectively. Treatment of MCF 7 cells with DOX/opt CH at a higher DOX concentration (100 µM), the percentages of viable cells after treatment for 24 h, 48 h and 72 h were 15%, 13%, and 7%, respectively. Similar results were obtained after treatment of MDA-MB-231 and FaDu cells with DOX/opt CH. Moreover, the results also showed a significant reduction in percentage cell viability (*p* < 0.005) for all cell lines treated with DOX/opt CH loaded with 50 μM of the drug for 24 and 48 h compared to the free drug solution (3.125 µM). This effect was also observed when the cells were treated with DOX/opt CH loaded with 25 μM of the drug for 72 h showing the ability of the CH to efficiently release the chemotherapeutic agent and exert an initial cytotoxic effect on treated cancer cells. 

Further assessment of the chemotherapeutic efficacy of DOX/opt CH was carried out by evaluating their IC_50_ compared to the free DOX solution in MCF 7, MDA-MB-231 cells and FaDu (Table 6). DOX/opt CH had higher IC_50_ compared to free DOX for all cell lines for the period of the treatment. However, it was also observed that the IC_50_ values of the DOX/opt CH were time-dependent and significantly decreased after 72 h of treatment (*p* < 0.005), confirming the ability of the optimal formulation to enhance the therapeutic efficiency of DOX and to decrease its toxicity and/or side effects. 

### 3.10. Cellular Uptake

**Figure 6:**
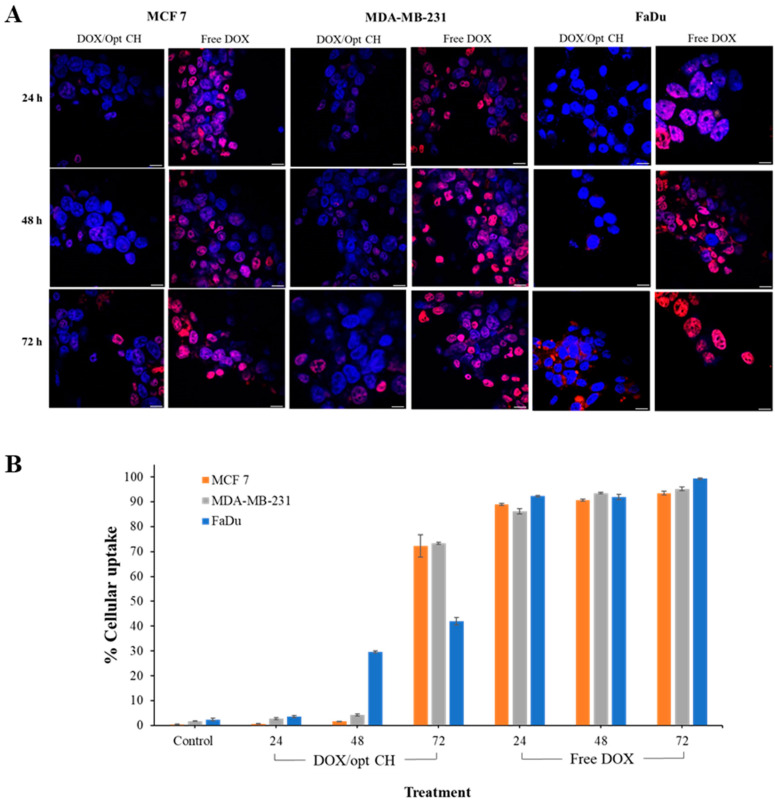
Cellular uptake studies in MCF 7, MDA-MB-231 and FaDu cells. (**A**) Qualitative analysis of DOX/opt CH and free DOX using fluorescent microscopy: in blue, the nuclei are stained with DAPI, while the red stain represents DOX (magnification 60×, scale bar = 30 μm). (**B**) Quantitative determination of % cellular uptake measured by flow cytometry. Data points are expressed as mean ± SD (n = 3).

The cellular uptake of the DOX/opt-CH was studied in MCF 7, MDA-MB-231 and FaDu cell lines after 24, 48 and 72 h of treatment via confocal microscopy (Figure 6A). The fluorescent micrographs showed a slight accumulation of DOX in all cell lines after 24 h of treatment with DOX/opt CH relative to the free drug solution. However, the cellular internalization of DOX considerably increased after 72 h of treatment with DOX/opt CH. The cellular uptake of DOX was also measured quantitatively using flow cytometry (Figure 6B). The results showed that the drug internalization in all cell lines was time-dependent, where the intracellular accumulation of DOX in FaDu cells increased significantly after 48 h of exposure. Treatment for 72 h showed a further significant increase in cellular uptake of DOX to levels approaching the free drug, especially in MCF 7 and MDA-MB-231cells indicating the ability of DOX/opt-CH in the vicinity of the cells to efficiently release the drug and facilitate its internalization.

The inhibition of cancer cell proliferation is a multifactorial process that might occur due to the accumulation/efflux of the cytotoxic agent and the genetic makeup of the cancer cells, among other factors [74,75]. The results indicate that the observed time-dependent decrease in cell viability could be due to an increase in the cellular uptake of DOX released from DOX/opt CH after prolonged exposure. DOX/opt CH was able to release the drug and initiate a therapeutic response in all studied cell lines. This effect can be possibly sustained over longer periods as the scaffold continues to release the drug slowly after local injection into the tumor site with minimal systemic side effects compared to the free drug. Further in vivo studies are still required to investigate the ability of DOX/opt CH scaffolds to release DOX and prevent malignant tumor progression after IT injection into solid tumors.

## 4. Conclusions

In this study, DOX-loaded GO/CS/GP CH scaffolds were prepared and optimized following a QbD approach that allowed for an effective analysis of the influence of formulation variables on the selected responses with a small number of experimental runs. The physicochemical characterization of the prepared formulations indicated the significant effect of the concentration of GO and CS as well as the CS:GP ratio on the gelation time, force required for injection, degree of porosity, and swelling capacity of the drug-loaded CH. The optimization process showed that the formulation prepared using 0.1% GO and 1.7% CS at a CS:GP ratio of 3:1 (*v/v*) had the highest desirability value. FT-IR analysis confirmed the lack of chemical interaction between DOX and other excipients used in the optimal formulation, while SEM imaging showed the porous microstructure nature of the DOX/opt CH. In vitro drug release studies showed that the incorporation of GO resulted in an increase in the ability of the hydrogel scaffold to control the release of DOX. Finally, treatment of different breast cancer and head and neck cancer cell lines with DOX/opt CH resulted in a time-dependent increase in cytotoxicity and cellular uptake of the drug and showed the biocompatibility of the DOX-free opt CH scaffolds. The proposed DOX/opt CH formulation can be considered a promising injectable platform for the IT administration of DOX for the treatment of solid tumors. Further investigations using suitable animal models are still required to determine the efficacy, biodistribution, biocompatibility, and pharmacokinetic parameters of DOX/opt CH in vivo. 

## Figures and Tables

**Table 1:** The studied independent variables (factors) and measured responses for the optimization of DOX-loaded GO/CS/GP CH.

Numerical Factors (Continuous)		Applied Levels	
		Low (−1)	High (+1)
X_1_	GO concentration (%*w/v*)	0	0.1
X_2_	CS concentration (%*w/v*)	1.5	2
**Numerical Factor (Discrete)**		**Applied Levels**	
X_3_	CS:GP ratio (*v/v*)	2:1	3:1
**Responses (Units)**		**Optimization Goal**	
Y_1_	Gelation time (min)	3 min	
Y_2_	Force required for injection (N)	Minimize	
Y_3_	Degree of porosity (%)	Minimize	
Y_4_	Swelling capacity (%)	Maximize	

**Table 2:** Experimental design and measured responses for the optimization of DOX-loaded GO/ CS/GP CH. X_1_: GO concentration, X_2_: CS concentration, X_3_: CS to GP ratio, Y_1_: gelation time, Y_2_: force required for injection, Y_3_: degree of porosity, Y_4_: swelling capacity.

Experimental Run	X_1_ (% *w/v*)	X_2_ (%*w/v*)	X_3_ (Ratio *v/v*)	Y_1_ (min)	Y_2_ (N)	Y_3_ (%)	Y_4_ (%)
F1	0.1	2	2:1	1.36 ± 0.15	5.69 ± 0.33	99.77 ± 11.23	129.11 ± 12.11
F2	0.1	1.75	3:1	2.87 ± 0.11	6.19 ± 0.14	112.74 ± 9.85	294.56 ± 15.69
F3	0.05	1.75	2:1	1.62 ± 0.42	5.96 ± 0.13	63.77 ± 7.43	96.84 ± 12.60
F4	0	1.5	2:1	32.67 ± 0.57	4.84 ± 0.30	78.47 ± 7.77	47.46 ± 6.67
F5	0.1	1.5	2:1	4.61 ± 0.11	4.26 ± 0.22	139.06 ± 8.43	46.55 ± 5.12
F6	0.1	1.75	3:1	2.98 ± 0.39	6.02 ± 0.25	106.98 ± 11.59	301.38 ± 24.50
F7	0.1	1.5	2:1	4.53 ± 0.06	4.07 ± 0.26	144.95 ± 7.30	41.27 ± 4.53
F8	0.05	2	2:1	0.30 ± 0.03	6.40 ± 0.16	82.74 ± 9.81	127.42 ± 12.15
F9	0	1.5	2:1	36.67 ± 0.57	4.84 ± 0.30	78.47 ± 7.77	47.46 ± 6.67
F10	0.05	1.75	3:1	0.23 ± 0.02	5.91 ± 0.33	62.87 ± 16.73	317.46 ± 30.77
F11	0.05	1.5	3:1	16.51 ± 0.34	2.43 ± 0.09	246.12 ± 31.02	129.22 ± 12.45
F12	0.05	1.5	2:1	9.06 ± 1.29	5.82 ± 0.67	79.32 ± 9.27	106.33 ± 12.53
F13	0	1.75	3:1	3.70 ± 1.30	7.01 ± 0.36	106.04 ± 10.59	189.02 ± 14.76
F14	0.05	1.5	3:1	16.36 ± 0.23	2.52 ± 0.10	251.44 ± 21.91	133.87 ± 14.98
F15	0.1	2	2:1	1.24 ± 0.12	5.82 ± 0.67	101.98 ± 12.66	121.36 ± 17.61
F16	0	2	2:1	0.51 ± 0.08	7.94 ± 0.48	101.91 ± 12.95	86.29 ± 14.98
F17	0	2	3:1	0.80 ± 0.39	10.10 ± 0.51	73.00 ± 12.52	170.83 ± 20.60
F18	0.05	2	3:1	0.17 ± 0.02	7.74 ± 0.62	79.25 ± 6.89	335.66 ± 19.52
F19	0	1.75	2:1	2.42 ± 0.18	6.12 ± 0.27	107.53 ± 16.12	49.58 ± 7.02

Data are mean values ± SD (n = 3).

**Table 3:** Quality target product profile (QTPP) of DOX-loaded GO/CS/GP CH.

QTPP Element		Target	Justification
Dosage form		Injectable hydrogel	In situ drug delivery with long residence time
Dosage design		Hydrophilic matrix	Sustain drug delivery with maximum biocompatibility and biodegradability
Route of administration		Intratumoral	Site-specific delivery and minimal systemic toxicity
Dosage product quality attributes	Physical attributes	Appearance	Sol-to-gel transition at body temperature for drug entrapment and controlled drug release
	Performance attributes	Gelation time	Optimal for proper handling and controlling the drug release
		Force for injection	Ease of injection into the solid tumor
		Porosity and swelling capacity	Control water uptake and drug release
	Chemical attributes	Identification	Study possible chemical interactions between the ingredients of the formulation

**Table 4:** **The** output results of the experimental design.

Response	Model Equation (*p*-Value)	R^2^	Adj-R^2^	Pred-R^2^	Adequate Precision	Significant Terms
Gelation time (min)	Quadratic (*p* = 0.0014)	0.947	0.912	0.845	16.98	X_1_ (*p* = 0.002) X_2_ (*p* < 0.0001) X_1_X_2_ (*p* < 0.0001) X_1_X_3_ (*p* = 0.0236) X_1_^2^ (*p* = 0.0342) X_2_^2^ (*p* = 0.0003)
Force for injection (N)	2FI (*p* = 0.001)	0.903	0.875	0.799	20.533	X_1_ (*p* = 0.0078) X_2_ (*p* < 0.0001) X_2_X_3_ (*p* < 0.0001)
Degree of porosity (%)	Quadratic (*p* = 0.0344)	0.863	0.795	0.687	12.193	X_2_ (*p* < 0.0001) X_3_ (*p* = 0.0064) X_2_X_3_ (*p* < 0.0001) X_2_^2^ (*p* = 0.0061)
Swelling capacity (%)	Reduced Quadratic (*p* = 0.0384)	0.922	0.883	0.771	14.847	X_1_ (*p* = 0.001) X_2_ (*p* = 0.0001) X_3_ (*p* < 0.0001) X_1_X_3_ (*p* = 0.0043) X_2_X_3_ (*p* = 0.0124)

**Table 5:** Predicted and observed values of the responses of the optimal formulation (DOX/opt CH).

Formulation Variables	Values	Responses	Predicted Values	Observed Values
X_1_	0.1%	Y1	3 min	1.18 ± 0.25 min
X_2_	1.7%	Y2	4.79 N	4.03 ± 0.32 N
X3	3:1	Y3	135.71%	141.32 ± 54.89%
		Y4	282.71%	311.31 ± 67.55%

**Table 6:** IC_50_ values (μM) for and the optimal formulation (DOX/opt CH in comparison with the free DOX solution.

Cell Type	DOX/opt CH			Free DOX Solution		
	24 h	48 h	72 h	24 h	48 h	72 h
MCF 7	19.24 ± 1.22	6.47 ± 0.51	2.50 ± 0.19	1.64 ± 0.09	1.52 ± 0.09	1.48 ± 0.11
MDB-MB-231	9.06 ± 0.78	6.13 ± 0.62	2.55 ± 0.17	2.33 ± 0.14	1.67 ± 0.14	1.53 ± 0.12
FaDu	14.67 ± 0.91	6.78 ± 0.43	3.03 ± 0.24	2.75 ± 0.18	1.21 ± 0.06	1.08 ± 0.08

## Supplementary Materials

The following supporting information can be downloaded at: https://www.mdpi.com/article/10.3390/nano12234261/s1, Figure S1. A standard calibration curve of DOX in phosphate buffer (pH = 7.4).
