# Supplementary material for: Graphene Oxide/Chitosan Injectable Composite Hydrogel for Controlled Release of Doxorubicin: An Approach for Enhanced Intratumoral Delivery
Source: Nanomaterials (Basel). 2022 Nov 30;12(23):4261. doi: 10.3390/nano12234261 (PMC9736459; doi:10.3390/nano12234261)
Supplement: Supplementary file 1 [file nanomaterials-12-04261-s001.zip › nanomaterials-2037592-supplementary.pdf]

nanomaterials

Supplementary Material

Graphene Oxide/Chitosan Injectable Composite Hydrogel for Controlled Release of Doxorubicin: An Approach for Enhanced Intratumoral Delivery

Safaa Eltahir [1], Reem Al homsi [1], Jayalakshmi Jagal [2], Iman Saad Ahmed [1,2] and Mohamed Haider [1,2,*]

1 Department of Pharmaceutics and Pharmaceutical Technology, College of Pharmacy, University of Sharjah, Sharjah 27272, United Arab Emirates
2 Research Institute of Medical & Health Sciences, University of Sharjah, Sharjah 27272, United Arab Emirates
* Correspondence: mhaider@sharjah.ac.ae; Tel.: +971-65057414

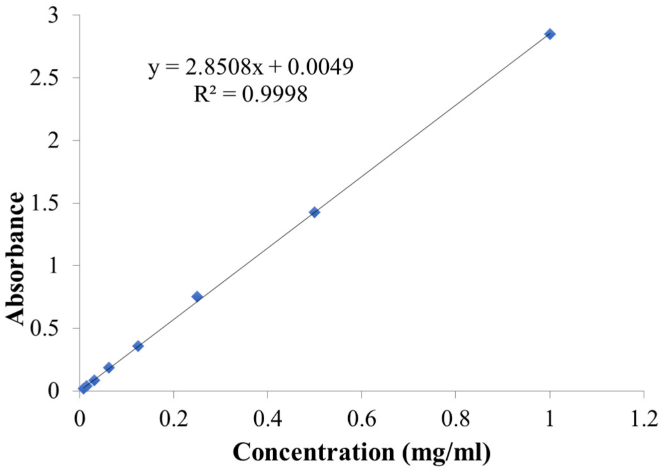

Figure S1. A standard calibration curve of DOX in phosphate buffer (pH = 7.4) was generated over the range of (0.001–1 mg/mL) using UV spectroscopy at 480 nm.

Nanomaterials **2022**, *12*, 4261. https://doi.org/10.3390/nano12234261 www.mdpi.com/journal/nanomaterials
